# Mining Twitter to Assess the Public Perception of the “Internet of Things”

**DOI:** 10.1371/journal.pone.0158450

**Published:** 2016-07-08

**Authors:** Jiang Bian, Kenji Yoshigoe, Amanda Hicks, Jiawei Yuan, Zhe He, Mengjun Xie, Yi Guo, Mattia Prosperi, Ramzi Salloum, François Modave

**Affiliations:** 1 Health Outcomes & Policy, University of Florida, Gainesville, Florida, United States of America; 2 Department of Computer Science, University of Arkansas at Little Rock, Little Rock, Arkansas, United States of America; 3 Department of Electrical, Computer, Software & Systems Engineering, Embry-Riddle Aeronautical University, Daytona Beach, Florida, United States of America; 4 School of Information, Florida State University, Tallahassee, Florida, United States of America; 5 Department of Epidemiology, University of Florida, Gainesville, Florida, United States of America; University of North Carolina at Charlotte, UNITED STATES

## Abstract

Social media analysis has shown tremendous potential to understand public's opinion on a wide variety of topics. In this paper, we have mined Twitter to understand the public's perception of the Internet of Things (IoT). We first generated the discussion trends of the IoT from multiple Twitter data sources and validated these trends with Google Trends. We then performed sentiment analysis to gain insights of the public’s attitude towards the IoT. As anticipated, our analysis indicates that the public's perception of the IoT is predominantly positive. Further, through topic modeling, we learned that public tweets discussing the IoT were often focused on business and technology. However, the public has great concerns about privacy and security issues toward the IoT based on the frequent appearance of related terms. Nevertheless, no unexpected perceptions were identified through our analysis. Our analysis was challenged by the limited fraction of tweets relevant to our study. Also, the user demographics of Twitter users may not be strongly representative of the population of the general public.

## Introduction

The Internet of Things (IoT) is receiving much attention as the number of connected devices to the Internet is projected to exceed 50 billion by the year 2020 [1]. Many see the IoT as a tremendous business opportunity. The term ‘IoT’ is relatively new, but is closely related to the old concepts of ubiquitous computing (also called pervasive computing). The words ubiquitous and pervasive mean “existing everywhere”, and are referring to the growing trend towards embedding microprocessors in everyday objects to make them completely connected and constantly available. Nevertheless, the IoT emphasizes the network of these everyday objects and their connectivity to the Internet. Although Mark Weiser from Xerox PARC is often credited for being the father of ubiquitous computing [2], the notion of “everywhere computing of connected end devices” is due to Ken Sakamura. Everywhere computing describes the spread of computing devices throughout our society, and in our lives, on a very large scale. This vision of Ken Sakamura in his TRON (The Real-time Operating system Nucleus) project [3] is expected to unfold as a consequence of the emerging IoT revolution.

On the other hand, social media platforms have become increasingly popular today. More people favor online social platforms to more traditional media sources for obtaining information in real time [4, 5]. As of January 2014, 74% of online adults use social networking sites [5]. Nevertheless, social media sites have served multiple purposes and functions with a wide diversity in content, including information dissemination, personal activities posting, product reviews, picture sharing, professional profiling, advertisements, political opinions, and sentiment. For example, networking services such as Facebook [6] are popular for users exchanging comments, pictures, videos etc. among their friends, whereas crowd-sourced review services such as Yelp [7] are conducive to sharing opinions about local businesses based on their personal experiences. Yet others like Twitter [8] are often used to disseminate status update of a person’s daily life, random thoughts, and personal opinions to the public. Consequently, businesses have access to largely untapped data from potential customers, and social media analysis has become an active multidisciplinary field, spanning domains such as computer science, behavioral psychology, mathematics, medicine, business analytics, and with as many different goals and outcomes.

Mining social media sites, Twitter in particular, has been the focus of numerous recent studies, with a broad range of focus: analyzing social media platforms to study how these platforms can facilitate smoking cessation [9], mining public health information [10], detecting influenza epidemics [11, 12], predicting election voting results [13], and studying global mood patterns [14]. In our previous studies, we have also mined Twitter messages for the detection of drug-related adverse events [15], the assessment of the adequacy of gender identification terms on medical intake forms [16, 17], and the analysis of U.S. weekly trends in work stress and emotion [18]. Social media provide new data sources that significantly expand the range of what can easily be measured, and thus facilitate computational knowledge discovery.

In this paper, our main objective is to acquire a better understanding of social opinion as it pertains the IoT, and how it has evolved over the past few years. Platforms such as Twitter are particularly useful to extract information related to user’s attitudes and perceptions on a variety of topics. As a micro blogging platform, Twitter has an enormous and increasing number of users, reaching over 307 million active users in the third quarter of 2015. On Twitter, users publish short messages using 140 or fewer characters to “tweet” about their opinions on various topics, to share information, and to have conversations with their ‘followers’. In turn, we show that these tweets generate data sets that can lead to a better understanding of social opinion regarding the IoT and produce evidence of its social influences.

## Methods

**Fig 1:**
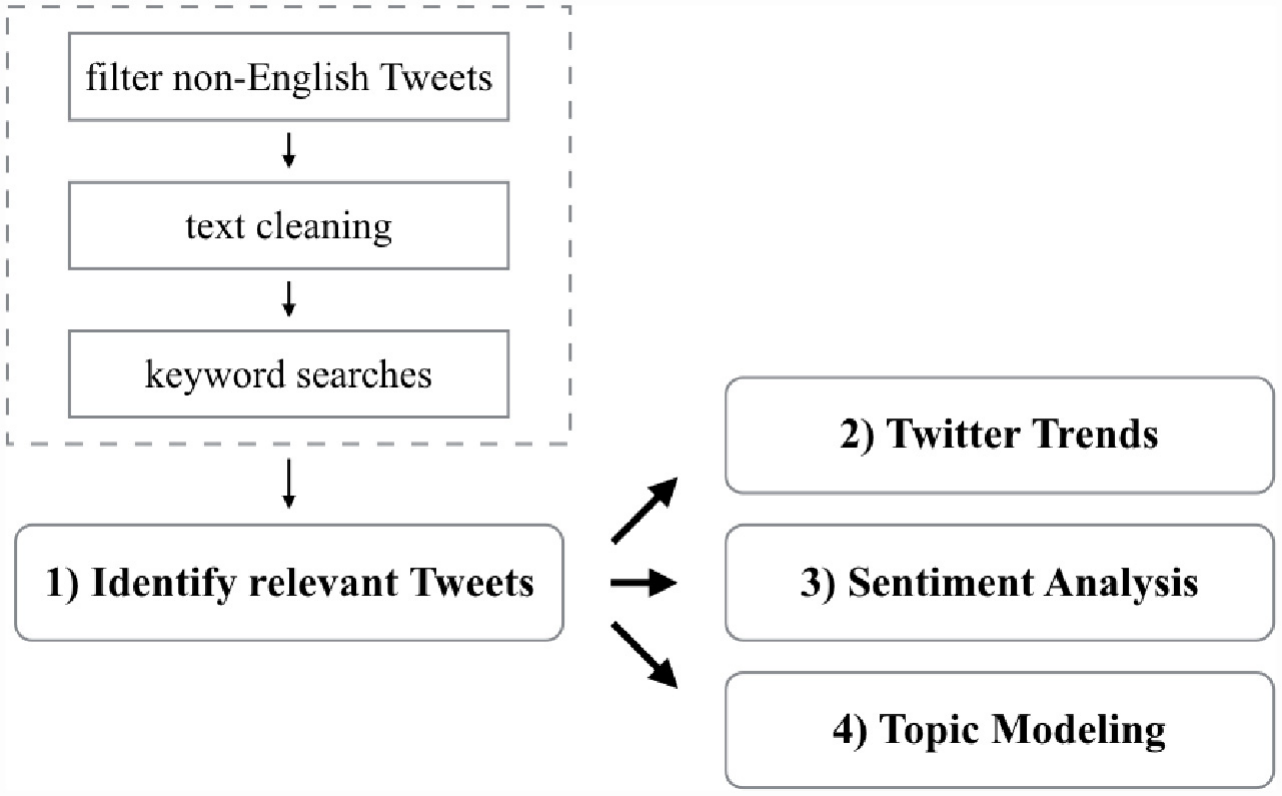
The analysis workflow consists of four main steps: 1) collect tweets that are potentially related to discussions about the IoT; 2) compose Twitter Trends to understand how the concepts of the IoTs have evolved; 3) assess public opinion with sentiment analysis; and 4) create structured information from tweets using topic modeling to find latent concepts relevant to the discussion of the IoT.

We leverage a number of different techniques to examine the various aspects of how the concept of IoT was developed and how its underlying emerging technologies impact the general public. The analysis workflow consists of four main steps, as depicted in Fig 1: 1) collect tweets that are potentially related to discussions about the IoT; 2) compose Twitter Trends to understand how the concepts of the IoTs have evolved; 3) assess public opinion with sentiment analysis; and 4) create structured information from tweets using topic modeling to find latent concepts relevant to the discussion of the IoT. We also utilize a number of visualization techniques to provide clear and easy-to-understand graphical representations such as word clouds to substantiate our findings. In the following sections, we describe each step and the basic procedures in further detail.

### Examining Trends in Twitter

Trend analysis is a common approach whereby one uses time series data to identify a pattern, or a trend. When examining a topic such as the IoT, trend analysis sheds light on when the specific concept emerged and helps us identify unusual patterns.

For instance, Google Trends (http://www.google.com/trends/) is a free and publicly available service that shows how often a particular search term is entered relative to the total search volume across time and in various regions of the world. For example, if we are interested in weekly trends, Google Trends produces relative search volume (RSV) indicators scaled to the week when the largest number of searches was conducted for that specific term (assigned an RSV = 100%). All other weekly search proportions are assigned RSV values relative to the highest search week for that term. By doing so, Google Trends normalizes the popularity of each topic, i.e., search term, between 0 and 100, thus making it possible to compare the trends of different search terms. Existing studies have provided evidence that Google Trends data can be used to effectively measure user behaviors as well as to monitor public health events such as outbreaks of influenza-like illness in a population [19]. Although the insights from such studies are valuable, we shall be aware of the limitations [20, 21, 22], and recognize the needs of using data from both traditional and these new sources.

We adapted the Google Trends methods to define Twitter trends with relative mention volume (RMV) indicators that show how often a particular concept is mentioned relative to the total volume of tweets across time. Based on a time unit of interest, e.g., weekly trends, we assign RMV = 100% to the largest number of weekly mentions for a specific concept; and the number of mentions of the same concept in all other weeks are converted to RMV values relative to the highest mention week. We will detail the process in the Results and Discussion section below.

### Understanding Public Opinion with Sentiment Analysis

There has been a significant amount of research on how sentiments are expressed in genres such as online reviews and news articles [23, 24]. Despite the constrained structure of language on Twitter, sentiment analysis on Twitter corpora has recently drawn significant attention [25, 26, 27, 28] as 1) the exploded popularity of social media and the sheer volume of the Twitter dataset make it a rich source of information; and 2) the Twitter data are readily available.

There are two general approaches to sentiment analysis: 1) a lexicon-based system such as Harvard General Inquirer [29], SentiWordNet [30], or Linguistic Inquiry and Word Count (LIWC) [31] which ties word choices to specific opinions or attitudes; and 2) a classification-based approach [32] that builds supervised classifiers from labeled instances of texts. The lexicon-based approach does not require any training data. However, it tends to be less accurate than the classification-based approach because of the inherent ambiguity in language.

Nevertheless, the drawback of the classification-based approach is that, in general, it is difficult to collect labeled datasets for the training process. When it comes to assessing the social influences of the IoT, a high degree of accuracy on sentiment analysis is not essential. Therefore, we apply a lexicon-based approach—LIWC—directly. Further, LIWC can assess a piece of text and then assign it to categories other than just a polarity sentiment (i.e., positive and negative emotions) with validated metrics.

### Discovery of Latent Topics through Topic Modeling

Topic models are statistical algorithms that can be used to discover the hidden thematic structure (i.e., topics) from large unstructured collections of documents by analyzing the words within the texts. One of the most significant features of topic models is that they do not require any prior annotations or labeling of the documents. The topics emerge from the analysis of the original texts, in part from words distribution in the documents. David Blei has an excellent introduction to probabilistic topic modeling published in the Communications of the ACM [33].

In this particular study, we apply the Latent Dirichlet allocation (LDA) [34], a generative probabilistic model, to categorize the collection of tweets into latent topics. In the LDA model, each document (e.g., tweet(s) in our study), treated as a vector of word counts using the bag-of-words approach, is viewed as a mixture of probabilities over the topics, where each topic is represented as a probability distribution over a set of words (i.e., the dictionary).

Before applying the LDA algorithm, we first sanitize the dataset by removing the hyperlinks, hash-tags "#" and reply-tags "@", and then apply the WordNet Lemmatizer in the Natural Language Toolkit (NLTK) [35] to group the different inflected forms of a word into its lemma. We also remove a list of stop words such as “a”, “I”, etc. and words that occur less than 3 times in our corpus since such infrequently found words do not construct meaningful latent topics in our context. By doing so, we create a "clean" dictionary for the LDA model.

## Results and Discussion

The motivation of this work is to shed lights on general public’s perception of the “Internet of Things” (IoT). We begin with a description of the data and delve in to a more in-depth discussion of model output. We have access to three different Twitter datasets. Two of these are historical datasets: one (~2 billion tweets) was collected from May 2009 to October 2010 by O’Connor et al [36], which we used previously to study drug-related adverse events [15]; and the other was collected from February 2014 to November 2014 by our colleagues at the University of Florida. Both are random samples of all public statuses posted in that time frame. We have also developed a Twitter crawler named *tweetf0rm* [37] and have been collecting random public tweets since January 2015. Overall, we have collected 2.96 billion tweets for this study during three time periods: May 2009 to October 2010, February 2014 to November 2014, and January 2015 to October 2015. Note that we have no data from late 2010 to early 2014. These datasets are not collected specifically for this study, but rather are existing datasets that we have access to. This is a general problem of collecting Twitter data, as Twitter does not provide access to historical data through their APIs, and active data collection is only allowed to random samples. Further, we filtered out non-English tweets using *langid* (https://github.com/saffsd/langid.py), a language identification tool. Out of the 2.96 billion tweets, 1.57 billion Twitter messages are written in English. These 1.57 billion tweets represent the corpus we used for analysis.

### A Twitter Crawler

We have developed a set of Python scripts leveraging the *twython* [38] library for accessing the Twitter APIs. We designed our Python crawler, *tweetf0rm* [37], to handle various potential runtime exceptions (e.g., the crawler will recover from a system failure automatically and pause collection when it reaches the Twitter API rate limits [39]) and distribute the workload across multiple Amazon EC2 instances. We verified the correctness of the data and the effectiveness of the crawler when we used it in a recent study to assess the adequacy of gender identification terms through mining Twitter [16] where we collected more than 154 million tweets over a 49-day period.

### Twitter Trends of IoT comparing to Google Trends

The search keywords that we used to define the trend of the IoT were variations of the word “Internet of Things” (e.g., “IoT”, and “InternetOfThings”) as well as their hashtag versions (e.g., “#IoT” and “#InterentOfThings”). Using these keywords only will ensure that we retrieve only tweets relevant to the discussion of the IoT. It is possible to include more search keywords such as product names (e.g., Nest), and concepts (e.g., Connected devices, and Smarthome) to enlarge the corpora. Nevertheless, English words are ambiguous in nature. For example, the word “nest” can mean either “bird nest” or “Goolge Nest”. Thus, the tweets retrieved by the keyword “nest” are not necessarily relevant.

**Fig 2:**
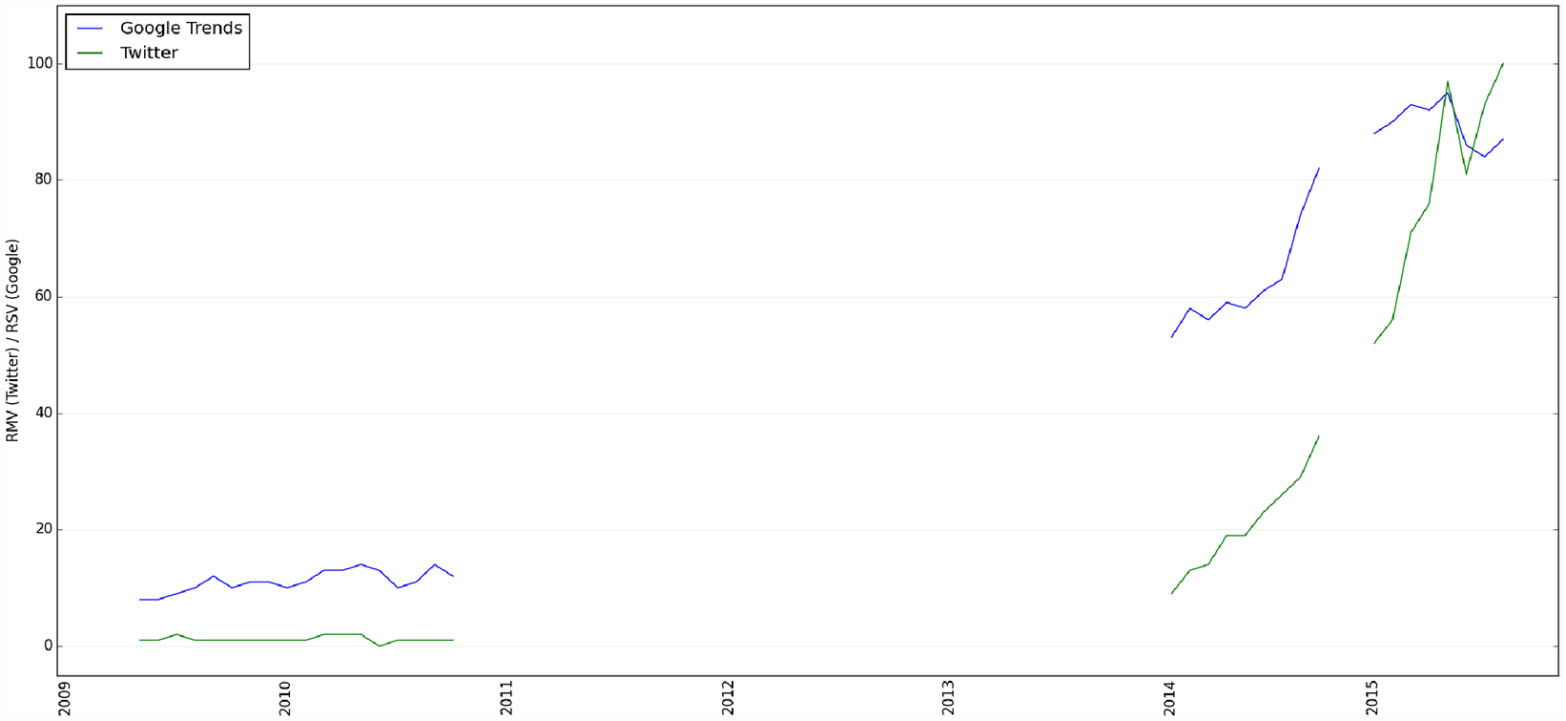
Trends on discussion of "Internet of Things" since 2009 in Twitter vs. Google Trends. For Google Trends, the y-axis is the relative search volume (RSV), and for Twitter trends, the y-axis is the relative mention volume (RMV).

Out of the 1.57 billion English tweets, we found 30,454 tweets containing one or more of these keywords. Fig 2 shows the monthly trends of discussing “Internet of Things” in Twitter (shown in green line) and compares it with the trending of the IoT on Google Trends (shown in blue line) since May 2009. The monthly Twitter mentions is normalized by the total number tweets of the respective month. The trends pertaining to the IoT in Twitter is highly correlated with the trends of the IoT on Google Trends (i.e., the Pearson correlation coefficient of all data points was 0.89 with a p-value of 1.86 × 10^−46^). These results validated our methods: Twitter is a quality resource comparable to Google search results in assessing trending discussions, at least when it comes to the IoT. Although it needs to be evaluated over a broad range of topics, this suggests that users express their opinions and explore topics in the same or similar timeframe regardless of whether they use Twitter or Google search.

**Fig 3:**
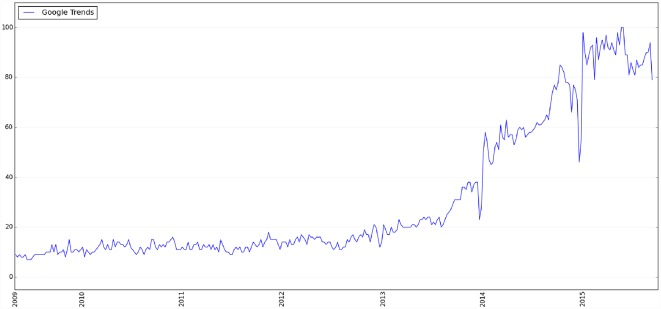
Google Trends of the search term "Internet of Things" since 2009.

Both the Google Trends and our Twitter trends shown in Fig 2 suggest a spike of interest in the “Internet of Things” in early 2014. However, we miss 2011, 2012, and 2013 data in our Twitter datasets. This raises the question of whether the spike actually happened on Twitter in 2014. Since we have already shown that Twitter trends analysis and Google Trends are strongly correlated, we plotted the national search trends of the IoT using weekly Google Trends data with no missing information since 2009. As shown in Fig 3, the search volume for the IoT was relatively low from 2009 to 2013; and a significant spike on Google Trends was evident around early 2014. Therefore, it is very likely that the original peak of interest in the IoT is indeed early 2014, and is evident by our Twitter trends analysis. Further, after examining the relevant tweets during this time period, we also found that many of the tweets are related to Google’s new smart home product, Nest, such as:

- “Great consumer tech at the #idealhomeshow this year with internet of things like webeye & nest. My fav micro drone 2.0”

And other industry leaders such as IBM, Cisco, and Apple also followed and connected their products with the IoT concept:

- “Cisco & AGT to roll out a suite of "smart cities" analytics technologies”
- “IBM, AT&T ink global Internet of Things tie-up”
- “SOLID conjecture around #Apple #iWatch IoT strategy: ecosystem, wears les, smart devices #kudos”

Many people also attempted to define the IoT, and discussed important concepts and different aspects of the IoT phenomenon, such as:

- “Cool infographic: A Guide to the Internet of Things http://t.co/SraYCPxrn2”
- “The Internet of Things Is Neither Dystopian Nor Utopian: http://t.co/wFhgYVY2i5”
- “Internet of Things requires new IT skills http://t.co/GWcuAg4t58 (interesting take on the human side of IoT) #iot #it”
- “Belkin #SmartHome networks in danger of hacks http://t.co/xDyfE1OyZd #Privacy #IoT #InfoSec #ConnectedHome”

We were able to discover many of these discussion themes using the topic modeling approach discussed below.

### Public sentiments towards the Internet of Things

**Fig 4:**
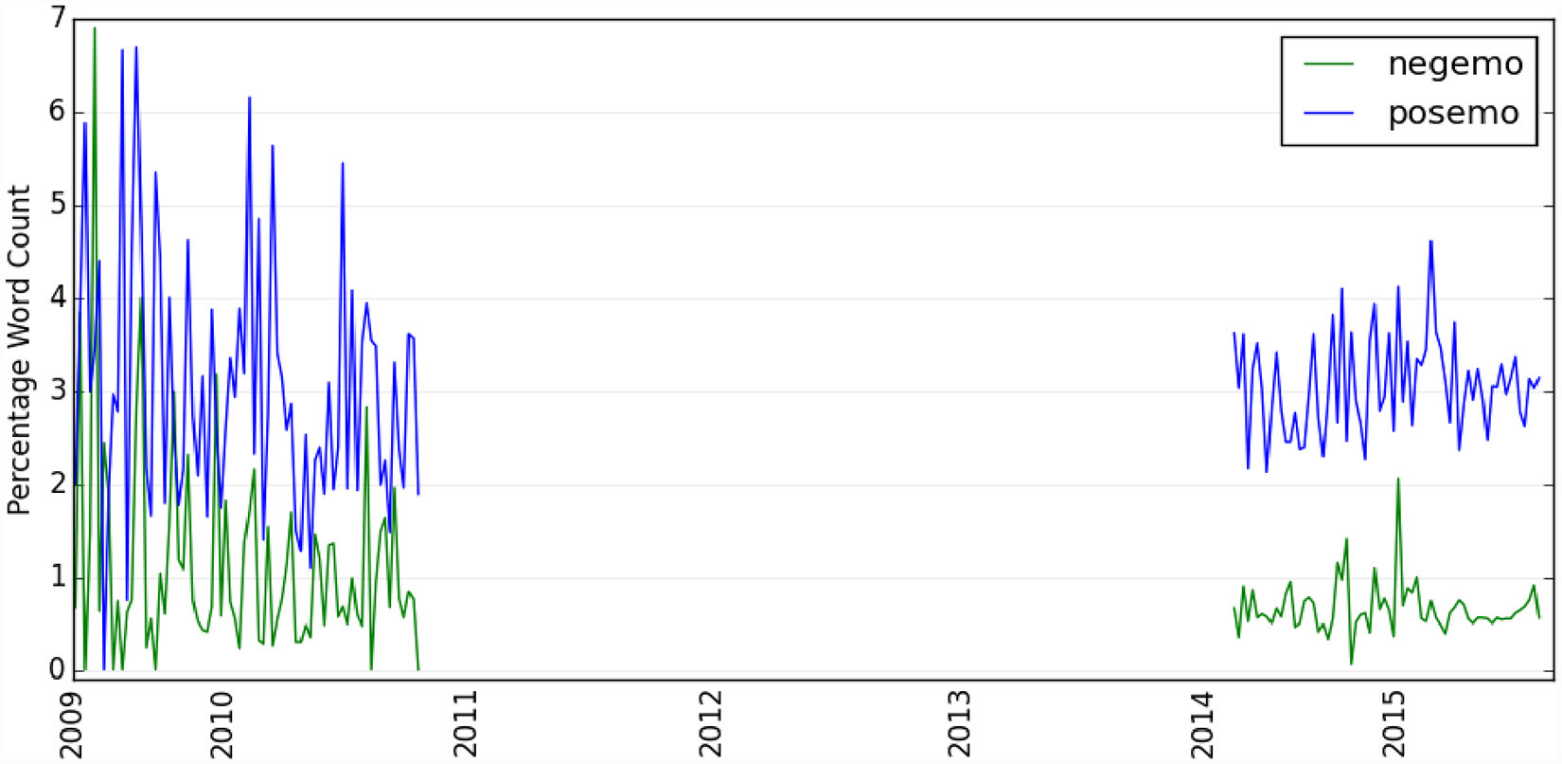
Public's emotion polarity towards Internet of Things since 2009: positive emotion vs. negative emotion. The y-axis is the percent of all the words in the text that are positive vs. negative emotion words.

**Fig 5:**
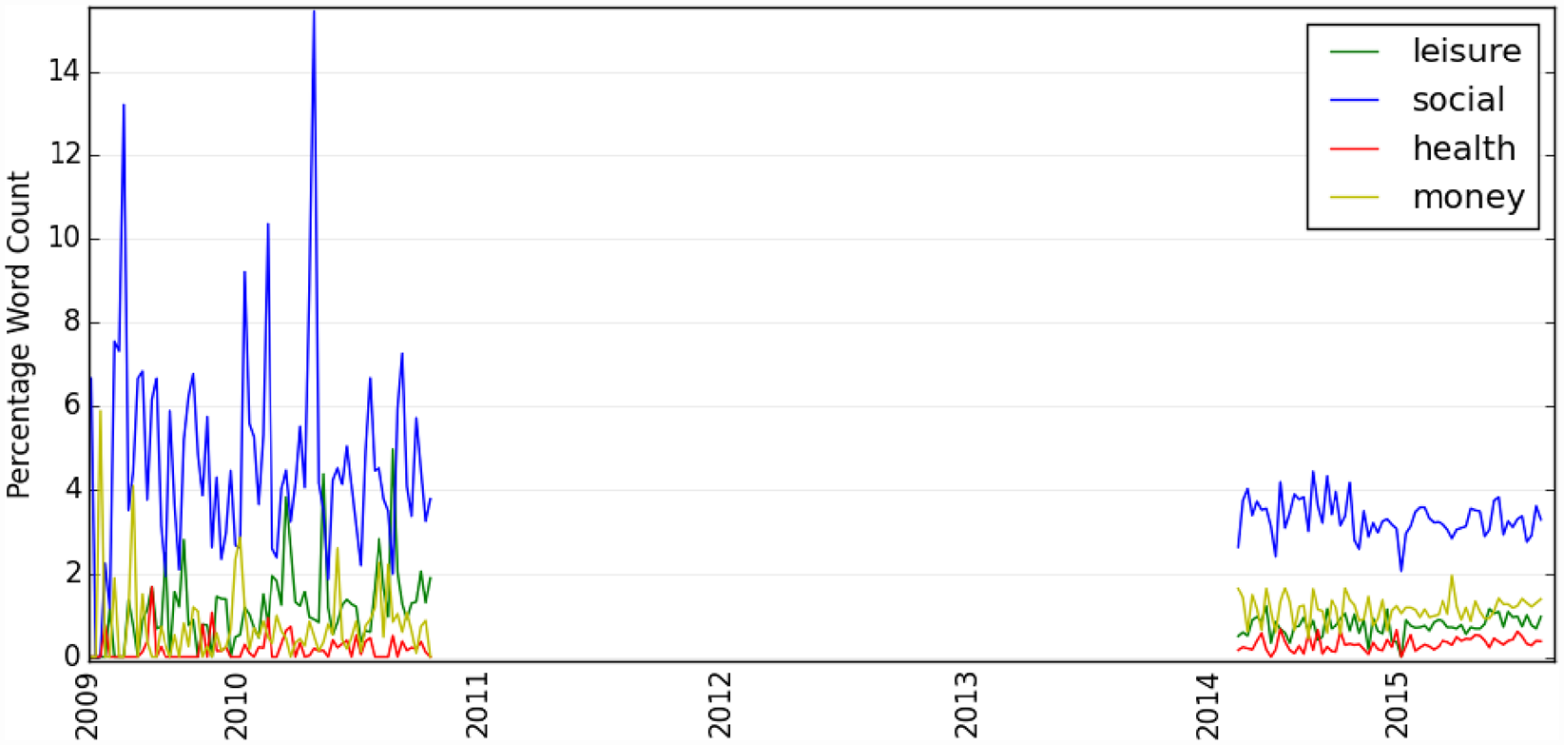
Public's sentiment on leisure, social, health and money when discussing Internet of Things. The y-axis is the percent of all the words in the text.

Through sentiment analysis using LIWC, we show (Fig 4) that Twitter users have expressed more positive than negative opinions about the IoT since 2009 with more fluctuations prior to 2014. These fluctuations are probably due to the smaller data samples available during that time period (i.e., between 2009 and 2010). Further, Fig 5 shows that, when people are discussing the IoT on Twitter, they are less likely to talk about aspects related to “*money”*, “*health”* and “*leisure”*, but are relatively more vocal on “*social”* issues (i.e., these sentiment concepts such as “money” and “social” are defined in LIWC). So, the Internet of Things might still just be a social function. As suggested by our data, the IoT might be another new buzzword like ‘big data’ that has been increasingly discussed online as a social phenomenon, but has not been materialized much in people’s daily activities. Nevertheless, it could also be the case that this result simply represents the nature of social media data, where discussions on social media platforms represent people’s social interactions (social processes) in nature.

Lexicon based sentiment analysis methods often raise concerns in terms of accuracy. To evaluate the accuracy of LIWC, we randomly selected 100 tweets and two reviewers evaluated the LIWC results (i.e., specifically, positive emotion vs. negative emotion). Each reviewer is asked to read the tweet and determine whether the tweet expresses a positive (1) or negative emotion (0). Note that LIWC provides scores for both positive and negative emotions rather than classify the tweet as either positive or negative. Many tweets express both positive and negative emotions, e.g., “It is exciting to see cars communicate with each other, but it might be dangerous for safety? #IoT #security”, where both LIWC positive and negative emotion scores are nonzero. In these cases, we consider the larger score as the final classification. For example, if a tweet’s positive emotion score is larger than the negative emotion, the tweet is classified as positive in the evaluation. The inter-rater agreement between the two raters is high (i.e., Cohen’s kappa, κ = 0.84). Out of the 100 random samples, LIWC classified 85 tweets accurately (i.e., accuracy: 85%).

### Latent topics related to the Internet of Things

**Fig 6:**
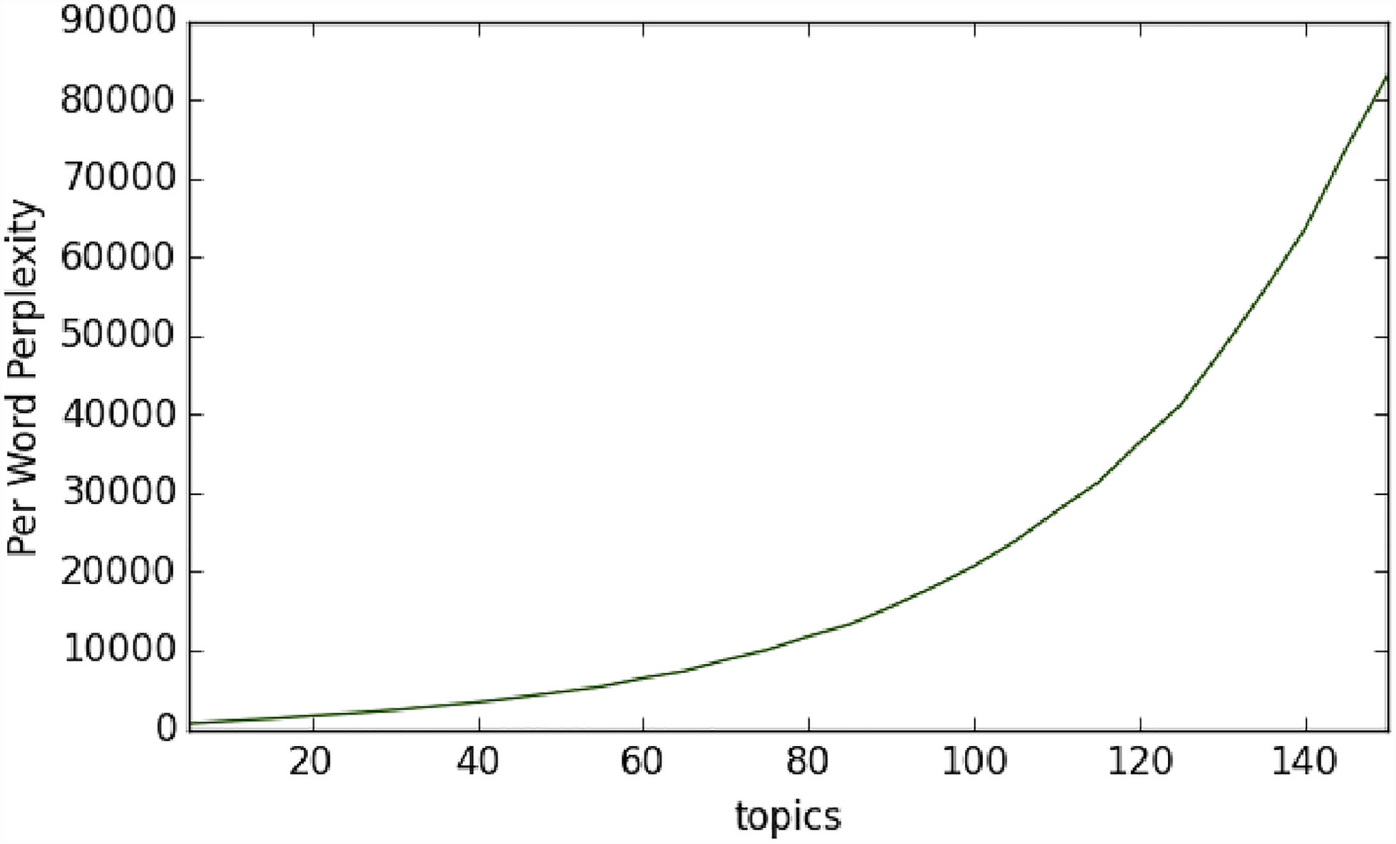
The perplexity score continues to increase as the number of topics increases.

One of the key parameters in LDA is the *number of topics*. A number of approaches have been proposed such as the Hierarchical Dirichlet process mixture model, which allows the number of topics to be unbounded and learned from data, and the nested Chinese Restaurant Process, which allows topics to be arranged in a hierarchical structure learned from data [40, 41]. Further, a number of hyper-parameters in the LDA model also need to be optimized based on the specific data [42, 43]. A common way to evaluate a probabilistic model such as LDA is to measure the log-likelihood of a held-out test set. Thus, we attempted to learn both the number of topics and the hyper-parameters through optimization of the *perplexity* on a held-out test set. However, Fig 6 shows that the per-word perplexity measure continues to increase when the number of topics increases. Thus, we failed to find the optimum number of topics through the perplexity score. On the other hand, not surprisingly, Chang et al [44] have shown that predictive likelihood (or equivalently, perplexity) and human judgment of the quality of learned topics are often not correlated. Thus, we manually evaluated the topics learned by varying the number of topics and decided that *15* topics are sufficient to describe our data regarding the IoT.

**Fig 7:**
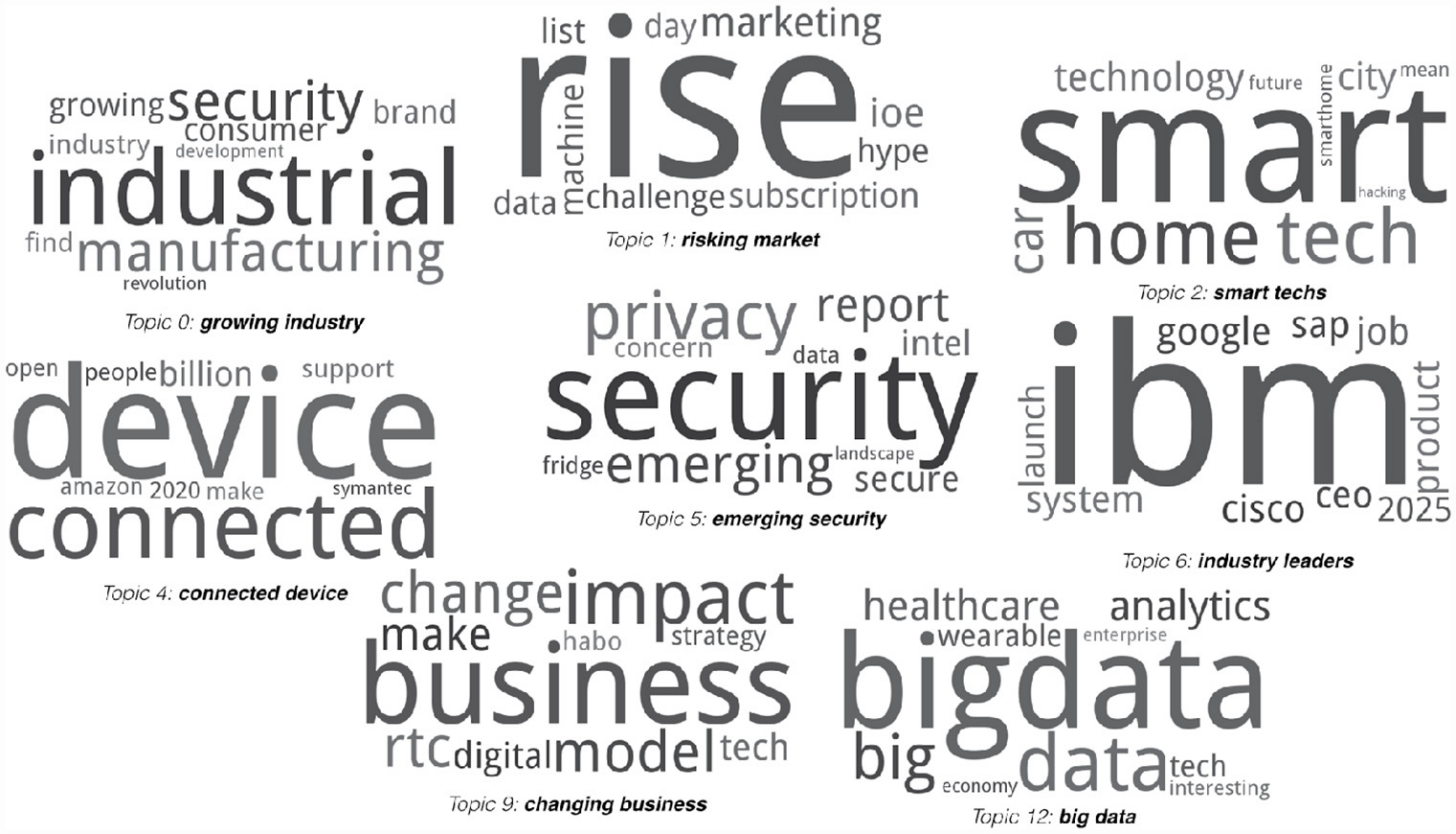
Topics learned from Twitter regarding the “Internet of Things”.

Since we learned probability distributions of words per topic (and a probability distribution of these topics over the entire collection of documents (tweets)) through LDA, each topic can be naturally visualized as word clouds where dominant words (i.e. words with high probabilities in each topic) are enlarged proportional to their probabilities in the topic. Fig 7 illustrates as word clouds the various concepts (topics) we learned from our Twitter datasets. The topic labels such as ‘*growing industry*’, ‘*risking market*’, and ‘*connected device*’ are assigned manually based on human judgment. Many of these topics capture important aspects of the IoT. Thus, it is reasonable to conclude that topic modeling, in particular, the LDA model, is a viable approach to learn characteristics of a concept based on Twitter datasets.

Through topic modeling, we discovered concepts that are clearly relevant to the IoT such as ‘*smart technologies*’, ‘*connected device*’, and ‘*big data*’. We also found topics we had not expected such as ‘*emerging security*’ and ‘*industry leaders*’. Fig 7 shows 8 word clouds out of the 15 topics learned from our Twitter datasets, and the interpretation of these topics are detailed below. The word clouds for the rest 7 topics are included as supplementary materials (S1 Fig).

- Growing industry—The IoT promotes automation and (thus efficient industry workflow) through M2M and machine intelligence. Thus, discussion relating to “industrial” and “manufacturing” are expected.
- Risking market—The IoT has been one of industry’s buzzwords for the recent years because of its un-paralleled market growth, and the related business opportunities have been publicized everywhere. Hence, it is quite understandable that if public would have a caution seen by “hype” for too many of optimistically forecasted business growth information.
- Smart technologies—Smart home systems are already on the market, and production and advancement of smart cars have led the automobile industry. Many highly dense cities are undertaking smart city projects. Based on the top words used in this topic, public opinions seem positive for smart technology, but there is a concern apparent by the term “hacking”.
- Connected device—The scale and connectivity of the IoT is particularly expressed in this topic (e.g., “connected”, “billion”, and “people”). Nevertheless, security seems to be a concerns as expressed by “Symantec” (a technology company who provides security products and services), possibly because of the “open”-ness of “connected” “device(s)”.
- Emerging security—As seen in this topic (and in previous topics already), “security” and “privacy” seems to be a great concern for the public. At the same time, the topic term “emerging security” also implies that some discussions were about positive opinions about the IoT to fix existing security concerns.
- Industry leaders—Several companies seem to be suggested as the IoT industry leaders. As observed from the data, the general public may be associating IoT more with a range of fortune 500 companies such as Amazon, Cisco, Google, and IBM, however, this may be almost always the case for most emerging technologies. This topic has dominated the discussion, perhaps due to excessive promotion of the technology by these companies.
- Changing business—The public seems to believe that the IoT can change their business supported by positive terms like “change” and “impact”.
- Big data—Big data in the IoT arises from information generated by wearable devices, and many applications of the IoT in the healthcare sector produce a vast amount of heterogeneous data. The public seems to have knowledge and interests in these big data, especially on analytics.

Further, “data,” “security,” and related terms (e.g.,. “hacking,” “privacy,” and “concerns”) appear often across a wide range of topics learned from the Twitter dataset. This implies that the public expresses great concern about privacy and security towards the IoT technology and relevant policy.

**Fig 8:**
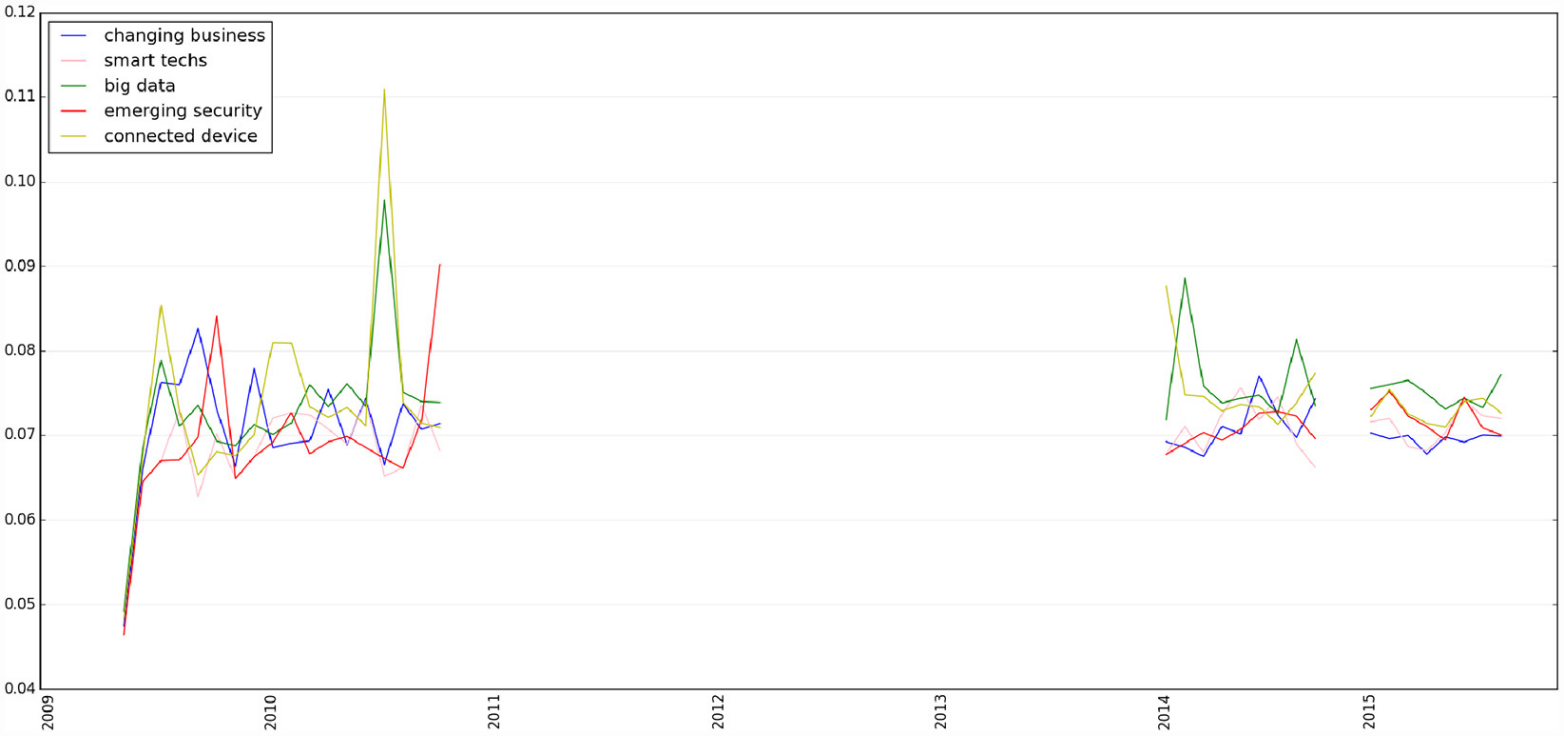
Trends of topics learned through a LDA model related to Internet of Things on Twitter since 2009. The y-axis is the probability distribution (between 0 and 1) of the topic of interest.

Furthermore, after constructing an LDA model, we can use the model to infer the distributions of topics given a piece of text. Fig 8 shows the trends of topics learned through a LDA model related to the IoT on Twitter since 2009, from which we can make a number of useful observations. For example, as shown in Fig 8, the concepts of ‘connected device’ and ‘big data’ raised significantly in late 2010 that are related to the surge in the number of mobile devices [45].

### Limitations

Our study shows that social media platforms such as Twitter can be used effectively to collect new data sets that can provide insight into emerging concepts and new technologies such as the Internet of Things. However, there are a number of limitations to our study.

First, in addition to the challenges associated with big data approaches in general, Twitter data not being restricted to a specific field may limit our ability to obtain data on topics that are rarely addressed in tweets. Although Twitter has a set of feature-rich APIs and a relatively open policy for scraping, collecting relevant data to answer a specific scientific question is not easy. For example, we collected over 3 years of data (2.9 billion raw tweets), however only a fraction of the data (30,454 tweets) was deemed relevant to our study.

Second, the demographics of Twitter users do not represent those of the general population. Indeed, we know that the users of social media tend to be younger (e.g., 37% of Twitter users are under 30, while only 10% are 65 years or older, as of 2014 [46]). Therefore, it may not be possible to generalize the findings on Twitter to the general population. Additionally, some Twitter users are deemed ‘power users’ and exhibit a substantially greater quantity of activity than the average user [5]. This may create a sample bias in the data. These issues need to be kept in mind for future studies using Twitter (or other social online platforms) for data mining and knowledge discovery.

Third, our analysis only focuses on general aspects of the IoT without considering the specific context of the discussion. For example, the public may exhibit different attitudes towards the same concept in different application fields of the IoT (e.g., safety in manufacturing vs. healthcare applications). The current study is exploratory, where we aim to investigate computational methods for exploring new big data sources such as Twitter. Such study, however, can point the directions of future in-depth studies to capture the richness of human perceptions.

## Conclusion

Given the ubiquitous nature of online social media platforms, and the amount of data they generate, they offer nearly unlimited and mostly untapped source of user-generated information, to assess user behavior, attitudes, and perceptions. Through Twitter data analysis, we discovered that the public’s perception on the Internet of Things is mostly positive for the period of our analysis (2009–2015). As they relate to the IoT, business and technology seem to be the main areas of interest. We did also find that the public expresses concern about privacy and security associated with the IoT. Though we had access to billions of raw tweets, our analysis was challenged by the limited fraction of the tweets relevant to our study. Also the user demographics available on the social media platforms do not allow our results to be generalized to the general population. Finally, we found that the IoT was not as a popular tweeting topic as we expected, presumably because the social media platform is more popular for younger generations who may not have much interest in the technology business in general. To our knowledge, this study is the first study on the public’s perception of the IoT based on social media data, and it provides us with valuable insight on a well-known subgroup of the general population, and thus may be used for future work with other social online platforms.

## Supporting Information

S1 Fig The other 7 topics learned from Twitter regarding the “Internet of Things”. (PNG)
