# Supplementary figures and images for: Mining Twitter to Assess the Public Perception of the “Internet of Things”
Source: PLoS One. 2016 Jul 8;11(7):e0158450. doi: 10.1371/journal.pone.0158450 (PMC4938510; doi:10.1371/journal.pone.0158450)

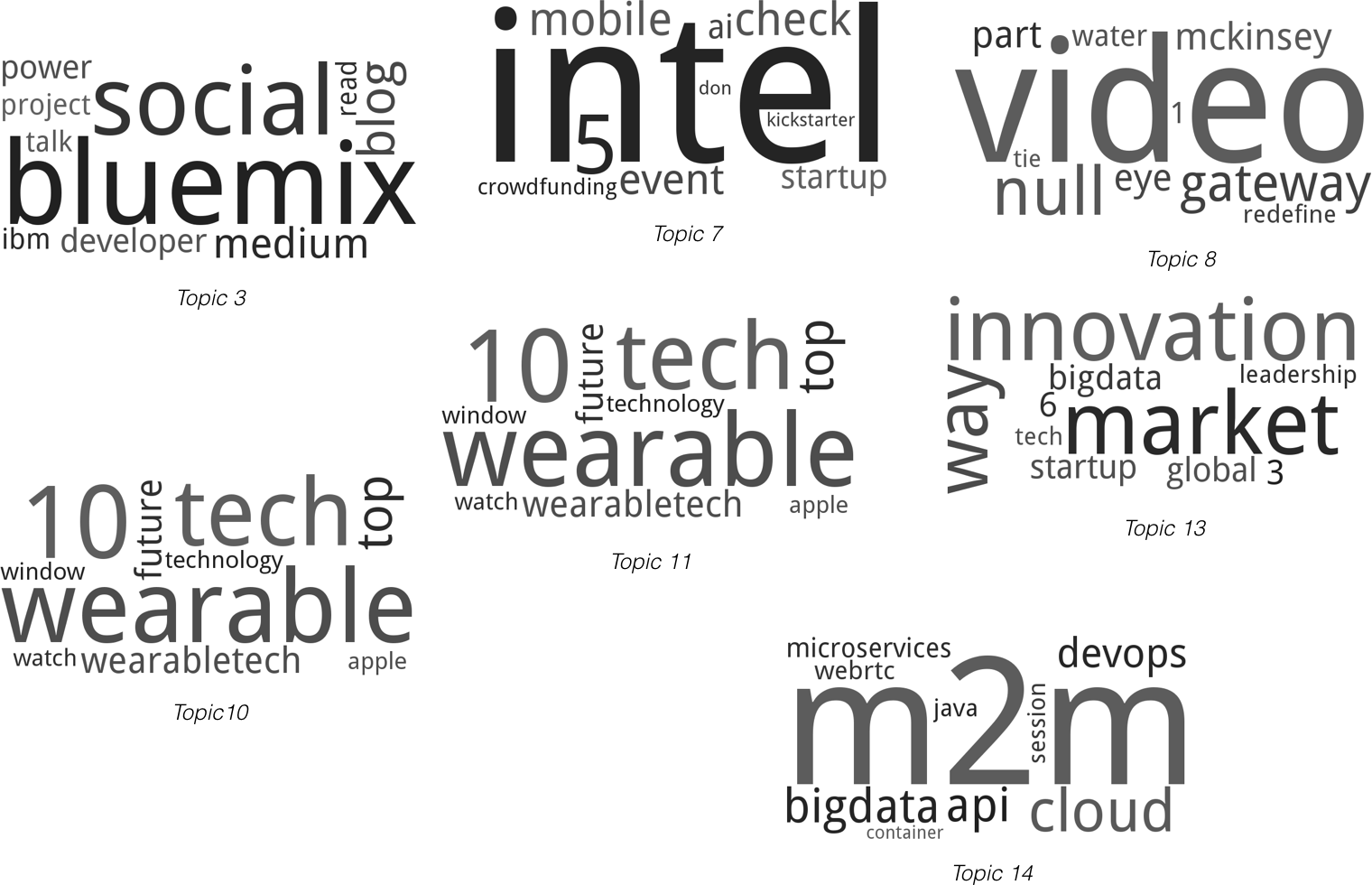

Supplement: S1 Fig — (PNG) [file pone.0158450.s001.png]
